# Intracranial haemorrhage without early clinical deterioration after mechanical thrombectomy: rethinking the “asymptomatic” label

**DOI:** 10.1093/esj/aakaf009

**Published:** 2026-01-01

**Authors:** Christoph Riegler, Christian H Nolte, Regina von Rennenberg, Kerstin Bollweg, Marianne Hahn, Timo Uphaus, Anna Alegiani, Till Illies, Johannes Wischmann, Lars Kellert, Kathleen Bernkopf, Silke Wunderlich, Florian Hennersdorf, Sven Poli, Leonhard Mann, Fee Keil, Ala Jamous, Marielle-Sophie Ernst, Franziska Bürkle, Martin Wiesmann, Burakhan Akkurt, Tobias Faizy, Heinrich J Audebert, Mike P Wattjes, Eberhard Siebert, Jawed Nawabi

**Affiliations:** Department of Neurology, Charité—Universitätsmedizin Berlin, Berlin, Germany; Center for Stroke Research Berlin (CSB), Charité—Universitätsmedizin Berlin, Berlin, Germany; Department of Neurology, Charité—Universitätsmedizin Berlin, Berlin, Germany; Center for Stroke Research Berlin (CSB), Charité—Universitätsmedizin Berlin, Berlin, Germany; Berlin Institute of Health (BIH), Charité—Universitätsmedizin Berlin, Berlin, Germany; Department of Neurology, Charité—Universitätsmedizin Berlin, Berlin, Germany; Center for Stroke Research Berlin (CSB), Charité—Universitätsmedizin Berlin, Berlin, Germany; Department of Neurology, Charité—Universitätsmedizin Berlin, Berlin, Germany; Center for Stroke Research Berlin (CSB), Charité—Universitätsmedizin Berlin, Berlin, Germany; Department of Neurology, University Medical Center Mainz, Johannes Gutenberg University, Mainz, Germany; Department of Neurology, University Medical Center Mainz, Johannes Gutenberg University, Mainz, Germany; Department of Neurology, Asklepios Klinik Altona, Hamburg, Germany; Department of Radiology and Neuroradiology, Asklepios Klinik Altona, Hamburg, Germany; Department of Neurology, University Hospital LMU Munich, Munich, Germany; Department of Neurology, University Hospital LMU Munich, Munich, Germany; Department of Neurology, School of Medicine Klinikum rechts der Isar, Technical University of Munich, Munich, Germany; Department of Neurology, School of Medicine Klinikum rechts der Isar, Technical University of Munich, Munich, Germany; Department of Diagnostic and Interventional Neuroradiology, University Hospital Tübingen, Tübingen, Germany; Department of Neurology and Stroke, Tübingen University Hospital, Tübingen, Germany; Hertie Institute for Clinical Brain Research, Tübingen University Hospital, Tübingen, Germany; Department of Neuroradiology, University Hospital Frankfurt, Goethe University, Frankfurt am Main, Germany; Department of Neuroradiology, University Hospital Frankfurt, Goethe University, Frankfurt am Main, Germany; Institute of Diagnostic and Interventional Neuroradiology, University Medical Center Göttingen (UMG), Georg-August-University Göttingen, Göttingen, Germany; Institute of Diagnostic and Interventional Neuroradiology, University Medical Center Göttingen (UMG), Georg-August-University Göttingen, Göttingen, Germany; Department of Diagnostic and Interventional Neuroradiology, University Hospital RWTH Aachen, Aachen, Germany; Department of Diagnostic and Interventional Neuroradiology, University Hospital RWTH Aachen, Aachen, Germany; Department of Radiology, Neuroendovascular Program, University Medical Center Münster, Münster, Germany; Department of Radiology, Neuroendovascular Program, University Medical Center Münster, Münster, Germany; Department of Neurology, Charité—Universitätsmedizin Berlin, Berlin, Germany; Center for Stroke Research Berlin (CSB), Charité—Universitätsmedizin Berlin, Berlin, Germany; Department of Neuroradiology, Charité—Universitätsmedizin Berlin, Freie Universität Berlin, Humboldt-Universität zu Berlin, Berlin, Germany; Department of Neuroradiology, Charité—Universitätsmedizin Berlin, Freie Universität Berlin, Humboldt-Universität zu Berlin, Berlin, Germany; Department of Neuroradiology, Charité—Universitätsmedizin Berlin, Freie Universität Berlin, Humboldt-Universität zu Berlin, Berlin, Germany

**Keywords:** ischaemic stroke, endovascular therapy, mechanical thrombectomy, (asymptomatic) ICH, haemorrhagic transformation

## Abstract

**Introduction:**

Intracranial haemorrhage (ICH) is a common complication following endovascular therapy (EVT) for ischaemic stroke. While symptomatic ICH (sICH) is known to worsen outcomes, the impact of ICH without early neurological deterioration (END), commonly referred to as “asymptomatic” (aICH), remains controversial. This study aimed to assess imaging patterns of aICH and its effect on clinical outcomes.

**Patients and methods:**

This study used data from the prospective, multicentre German Stroke Registry-Endovascular Treatment. Bleedings were assessed on follow-up imaging at 24 hours applying the Heidelberg Bleeding Classification. European Cooperative Acute Stroke Study III (ECASS)-III criteria were used to stratify patients into (1) no ICH, (2) aICH and (3) sICH. The primary outcome was functional independence (mRS ≤ 2) at 3 months. Secondary outcomes included mRS shift and 3-month mortality.

**Results:**

Among 4834 patients with EVT (median age 76, 51% female, median NIHSS 14), ICH occurred in 13.2% (aICH: 9.7%, sICH: 3.5%). Haemorrhage patterns differed, with sICH being more often parenchymal (48.2% vs 34.6%), multicompartmental (34.1% vs 20.2%) and involving the ventricular system (18.8% vs 7.6%), while aICH were predominantly haemorrhagic transformation (34.6% vs 21.8%). Functional independence at 90 days was reached by 40.0% (no ICH), 25.4% (aICH; adjusted odds ratio [aOR] 0.43 [0.32–0.58]) and 6.5% (sICH; aOR 0.06 [0.03–0.14]), respectively. aICH was associated with worse overall recovery (mRS shift adjusted common OR 0.51 [0.41–0.63]) and higher 90-day mortality (35.5% vs 24.9%; aOR 1.90 [1.44–2.51]), when compared to no ICH.

**Conclusion:**

ICH after EVT was associated with worse functional recovery and higher mortality, even in the absence of END. Given these results, the term “asymptomatic ICH” warrants reconsideration.

## Graphical Abstract

**Graphical abstract:**
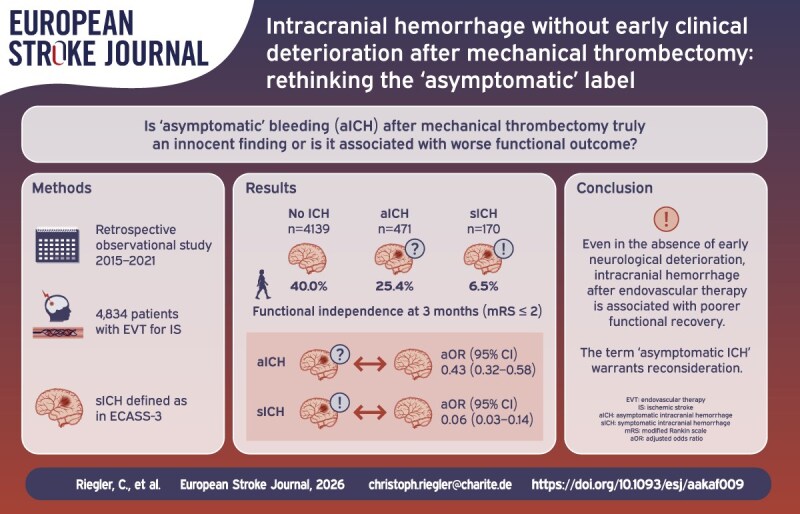

## Introduction

Acute ischaemic stroke due to large vessel occlusion is a leading cause of disability and mortality worldwide.^1^ Mechanical thrombectomy (MT) has revolutionised stroke treatment, offering significant improvements in functional outcomes and survival. However, intracranial haemorrhage (ICH) remains a well-documented complication of reperfusion therapies, occurring in up to 49% of patients post-thrombectomy.^2^ Symptomatic ICH (sICH) is widely recognised as a surrogate marker of poor outcomes and frequently used as a safety endpoint in stroke trials.^2–4^ Rates of sICH varied across stroke trials for MT, ranging from 4.4% in the Highly Effective Reperfusion Evaluated in Multiple Endovascular Stroke (HERMES) meta-analysis up to 7% in late-window trials and up to 7.8% in large core infarct trials.^3–9^ However, the clinical implications of ICH without prompt neurological deterioration, commonly referred to as “asymptomatic ICH (aICH)”, remain controversial.^10^^,^^11^

Historically, aICH—occurring in 30%–40% of patients—has been considered a benign radiological finding without functional consequences.^10^^,^^12^^,^^13^ However, emerging evidence challenges this assumption.^10^^,^^14^ Several studies have reported an association between aICH and worse long-term functional outcomes as well as an increased mortality, even in the absence of immediate neurological deterioration.^15–20^ The mechanisms underlying this association remain speculative but may involve neuroinflammation, blood–brain barrier dysfunction and delayed secondary brain injury.^21^

Recent meta-analyses, including the study by Harker et al. (2024), suggest that patients with aICH following MT have significantly higher odds of poor 90-day functional outcomes and increased mortality compared to those with no ICH.^10^ However, discrepancies in study design, imaging criteria and haemorrhage classification systems (eg, European Cooperative Acute Stroke Study [ECASS], Safe Implementation of Thrombolysis in Stroke-Monitoring Study [SITS-MOST], Heidelberg Bleeding Classification) contribute to heterogeneity in findings. The question remains: Should aICH be considered a relevant safety endpoint in thrombectomy trials, or does it merely reflect successful reperfusion?^12^ Understanding the true impact of aICH is crucial for refining risk stratification, optimising post-thrombectomy management and guiding future neuroprotective strategies.

Our study utilised a large national, multicentre prospective observational stroke registry to provide a comprehensive analysis of ICH phenotypes and their impact on functional outcomes. By employing a detailed classification approach, we aim to refine the prognostic significance of aICH in modern thrombectomy practice.

## Patients and methods

### Study population

The study was conducted inside the German Stroke Registry-Endovascular Treatment (GSR-ET), a national, multicentre, prospective observational registry, which methods have been previously detailed.^22^^,^^23^ The ongoing GSR-ET records data of all consecutive individuals receiving endovascular therapy (EVT) for ischaemic stroke in its participating centres since 2015. Functional status on the mRS at 3 months is regularly assessed. In the presented study, we included patients treated with EVT between 2015 and 2021. The GSR-ET comprises 25 academic and non-academic tertiary stroke centres. During the planning process, all centres were approached for participation. Ultimately, 8 academic centres and 1 non-academic centre consented to participate. All contributing centres thoroughly evaluated follow-up imaging at 24-hour follow-up (cerebral CT or MRI) to identify patients with ICH.

Whenever ICH was present, the Heidelberg Classification was applied to describe detailed bleeding patterns as follows^24^: class 1a: scattered small petechiae, class 1b: confluent petechiae, class 1c: parenchymal haematoma of less than 30% of the infarcted tissue; class 2: parenchymal haematoma of at least 30% of the infarcted tissue with obvious mass effect; class 3a: parenchymal haematoma outside the infarcted area, class 3b: intraventricular haemorrhage (IVH), class 3c: subarachnoid haemorrhage (SAH) and class 3d: subdural haemorrhage. Based on the ECASS-III criteria, we defined symptomatic ICH as any visible ICH associated with a neurological deterioration of ≥ 4 points on the NIHSS, in the absence of a more likely alternative explanation for the clinical worsening.^25^ We compared 3 groups of patients: (1) patients with no ICH; (2) patients with ICH without early neurological deterioration (END), referred to as “asymptomatic” (aICH); (3) patients with ICH and END, referred to as symptomatic (sICH). Baseline variables, procedural details and clinical outcomes were compared to assess the potential impact of aICH on clinical outcome.

### Variables and outcomes

We assessed stroke severity using the NIHSS. The *Alberta Stroke Program Early CT Score* (ASPECTS) was applied to assess early ischaemic changes on baseline imaging. Functional independence at 3 months (mRS ≤ 2*)* was defined as primary outcome.^26^ As secondary outcomes, we assessed dependency with unassisted ambulation (mRS ≤ 3; *fair outcome*) and disability at 3 months assessed through mRS shift analysis.^26^ Early neurological improvement (ENI), meaning a decrease of at least 4 points on the NIHSS between admission and 24 hours or reaching an NIHSS score of 0 at 24 hours was selected as a short-time clinical outcome.^27^ We further assessed an association of aICH and sICH with in-hospital mortality and mortality at 3 months.

The *modified thrombolysis in cerebral infarction* (mTICI) *scale* was used to evaluate technical EVT success.^28^  *Successful recanalisation* was reached whenever final mTICI was 2b or 3; *complete recanalisation* in case of mTICI 3. Procedural complications of interest were vasospasms, periprocedural clot migration and dissection/perforation.

### Statistical analysis

Treatment times and continuous baseline variables are presented as median (interquartile range [IQR]), binary variables as absolute numbers and percentages. Continuous and ordinal baseline variables were compared using the Kruskal–Wallis test (3-group comparison) and Mann–Whitney *U* test (2-group comparison aICH vs no ICH and sICH vs no ICH). Chi-square test was used for binary variables. Binary logistic regression models were conducted to assess an association between the occurrence of aICH or sICH with clinical outcomes (with no ICH as comparison group). All models were adjusted for age, sex, NIHSS at admission, pre-stroke dependency, intravenous thrombolysis, time between symptom onset (or last-seen-well) to hospital arrival, diabetes mellitus, arterial hypertension, atrial fibrillation, hyperlipidemia, current smoking, ASPECTS, successful recanalisation (mTICI 2b/3), antiplatelets and oral anticoagulation (at baseline, respectively). The analyses were conducted using IBM SPSS Statistics for Windows, Version 29.0.0.0, Armonk, NY: IBM Corp.^29^

### Informed consent and ethics approval

The GSR-ET registry received central approval from the Ethics Committee of the Ludwig-Maximilians University (LMU) in Munich (689-15).^30^ Quality assurance for patients undergoing EVT in Germany is mandated by federal law; thus, informed consent was not mandatory in accordance with local rules and regulations. This helped minimise selection bias by lack of informed consent.^31^

## Results

All GSR-ET patients receiving EVT between 2015 and 2021 with documented ICH and available Heidelberg Bleeding Classification were included in the study. The final study sample consisted of 4834 patients from 9 centres. At 24-hour follow-up imaging, 4139 patients (86.8%) had no ICH, 471 patients (9.5%) had aICH and 170 patients (3.5%) had sICH. Bleeding status was assessed using cerebral CT in most patients, MRI was used in 563 (13.6%) patients with no ICH, 79 (16.8%) patients with aICH and 8 (4.7%) patients with sICH (*P* < .001).

Haemorrhagic transformation (Heidelberg class 1a/1b) without any other bleeding was detected in 34.6% of patients with aICH and 21.8% of patients with sICH (*P* < .01). Parenchymal haematoma (PH; class 1c/2/3a) with or without intraventricular (3b) or subarachnoid (3c) component accounted for 34.6% of patients with aICH and 48.2% of patients with sICH (*P* < .01).

While PH restricted to the infarcted area (1c/2) occurred in similar frequency in aICH (22.1%) and sICH (22.9%), PH (1c/2) with any remote (3a), ventricular (class 3b) or subarachnoid (3c) component comprised only 10.0% of patients with aICH, but 21.2% of patients with sICH (*P* < .001). Remote PH (class 3a) occurred in 1.7% (aICH) and 2.4% (sICH) and remote PH (3a) plus other bleeding (1b, 3b/c/d) was found in 0.8% and 1.8% of patients. Isolated IVH (class 3b) occurred in 5 patients (3 aICH vs 2 sICH). Subarachnoid haemorrhage (class 3c) without PH comprised about 30% of both asymptomatic and symptomatic bleedings (*P* = .83).

Multicompartmental bleedings involving more than 1 Heidelberg class comprised about one third of sICH (34.1%), yet only one-fifth of aICH (20.2%, *P* < .001). Similarly, ICH including any intraventricular bleeding component (3b) was predominantly symptomatic (sICH: 19.7% vs aICH: 6.8%, *P* < .001). For more details on Heidelberg Bleeding Classification results, see Table 1 and Table S1 in the Supplementary material.

**Table 1:** Heidelberg Bleeding Classification—main groups.

	**All** ***n* = 641**	**aICH** ***n* = 471**	**sICH** ***n* = 170**	***P***
**Follow-up imaging**				
CT, *n* **(%)**	554** (86.4)**	392 **(83.2)**	162 **(95.3)**	**<.001**
MRI, *n* **(%)**	87 **(13.6)**	79 **(16.8)**	8 **(4.7)**	
**Heidelberg Bleeding Classification**, *n* **(%)**				
HT (1a or 1b)	200 **(31.2)**	163 **(34.6)**	37 **(21.8)**	**<.01**
PH (any)	245 **(38.2)**	163 **(34.6)**	82 **(48.2)**	**<.01**
PH only (infarcted tissue—class 1c/2)	143 **(22.3)**	104 **(22.1)**	39 **(22.9)**	.82
PH (1c/2) plus remote bleeding (3a/b/c/d)	83 **(12.9)**	47 **(10.0)**	36 **(21.2)**	**<.001**
Remote PH (class 3a only)	12 **(1.9)**	8 **(1.7)**	4 **(2.4)**	.59
Remote PH plus other bleeding (classes 1a/b, 3b/c/d)	7 **(1.1)**	4 **(0.8)**	3 **(1.8)**	.33
IVH (3b)	5 **(0.8)**	3 **(0.6)**	2 **(1.2)**	.49
SAH (3c; any)	189 **(29.5)**	140 **(29.7)**	49 **(28.8)**	.83
SAH only (3c)	129 **(20.1)**	97 **(20.6)**	32 **(18.8)**	.62
SAH (3c) plus HT (class 1a/b) or IVH (class 3b) or SDH (3d)	60 **(9.4)**	43 **(9.1)**	17 **(10.0)**	.74
SDH (3d)	2 **(0.3)**	2 **(0.4)**	0	.40
More than one class	153 **(23.9)**	95 **(20.2)**	58 **(34.1)**	**<.001**
Any intraventricular component (±HT, PH or SAH)	68 **(10.6)**	36 **(7.6)**	32 **(18.8)**	**<.001**

Abbreviations: HT = haemorrhagic transformation; IVH = intraventricular haemorrhage; PH = parenchymal haematoma; SAH = subarachnoid haemorrhage; SDH = subdural haemorrhage. *p* values <0.05 marked bold.

### Baseline demographics

Age and sex were equally distributed within the 3 groups. When compared to no ICH, stroke severity (NIHSS) was higher in aICH (median [IQR] 15 [11–19] vs 14 [8–18], *P* < .001) and patients with aICH had higher rates of diabetes mellitus (26.5% vs 20.6%, *P* < .01). Pre-medication with antiplatelets was more common in patients with aICH (32.1% vs 26.2% [no ICH], *P* < .01). All other baseline variables and stroke aetiology were equally distributed between aICH and no ICH. We found no differences between patients with aICH and no ICH regarding witnessed onset of stroke, time-to-hospital arrival and imaging modality at admission (CT/MRI). However, perfusion-based imaging was performed less often in patients with aICH (46.1% vs 54.8%, *P* < .001) and ASPECTS was lower in aICH (median [IQR] 8 [7–10]) than in no ICH (median [IQR] 9 [7–10]) patients. Compared to patients with no ICH, patients with aICH tended to have more proximal vessel occlusions, in particular carotid-T and proximal M1 segment of the MCA. Basilar occlusion was less common in patients with aICH (3.8% vs 9.7% [no ICH]). Detailed data on occlusion site and further baseline variables are presented in Table 1.

Patients with sICH differed from patients with no ICH in several baseline and procedural variables, predominantly more pre-stroke dependency, lower rates of hyperlipidemia, less perfusion-based imaging and lower ASPECTS. As this study focuses on aICH, more detailed information on sICH can be found in Table 2.

**Table 2:** Baseline demographics, treatment times, procedural data.

	**All** ***n* = 4,834**	**No ICH** ***n* = 4,139**	**aICH** ***n* = 471**	**sICH** ***n* = 170**	***P* (aICH vs no ICH)**	***P* (sICH vs no ICH)**	***P* (overall)**
Age, median (IQR)	76 (65–83)	76 (65–83)	76 (64–83)	76 (65–82)	.34	.52	.54
Female, *n* (%)	2,461 (50.9)	2,137 (51.0)	235 (49.9)	89 (52.4)	.66	.72	.84
Pre-stroke dependency (mRS > 2), *n* (%)	634 (13.4)	552 (13.4)	49 (10.5)	33 (19.6)	.08	**.02**	**.01**
Admission NIHSS, median (IQR)	14 (9–18)	14 (8–18)	15 (11–19)	14 (10–18)	**<.001**	.19	**<.001**
Smoking, *n* (%)	678 (14.0)	604 (14.4)	55 (11.7)	19 (11.2)	**.11**	.24	.15
Hypertension, *n* (%)	3,596 (74.4)	3,125 (74.5)	349 (74.1)	122 (71.8)	.84	.42	.71
Atrial fibrillation, *n* (%)	2,164 (44.8)	1,891 (45.1)	208 (44.2)	65 (38.2)	.70	.08	.20
Diabetes mellitus, *n* (%)	1,031 (21.3)	863 (20.6)	125 (26.5)	43 (25.3)	**<.01**	.14	**<.01**
Hyperlipidemia, *n* (%)	2,067 (42.8)	1,839 (43.9)	184 (39.1)	44 (25.9)	.047	**<.001**	**<.001**
Antiplatelets, *n* (%)	1,303 (27.0)	1,098 (26.2)	151 (32.1)	54 (31.8)	**<.01**	.11	**.01**
Oral anticoagulation, *n* (%)	1,067 (22.1)	946 (22.6)	88 (18.7)	33 (19.4)	.06	.34	.11
**Stroke aetiology**							
Larger-artery atherosclerosis, *n* (%)	944 (19.5)	811 (19.3)	100 (21.2)	33 (19.4)	.33	.98	.62
Cardioembolic, *n* (%)	2,515 (52.0)	2,199 (52.4)	239 (50.7)	77 (45.3)	.48	.07	.16
Dissection, *n* (%)	106 (2.2)	92 (2.2)	8 (1.7)	6 (3.5)	.48	.25	.38
Other determined, *n* (%)	261 (5.4)	215 (5.1)	28 (5.9)	18 (10.6)	.45	**<.01**	**<.01**
Undetermined, *n* (%)	1,008 (20.9)	876 (20.9)	96 (20.4)	36 (21.2)	.80	.93	.96
**Treatment times**							
Witnessed onset of stroke, *n* (%)	2,801 (57.9)	2,247 (58.4)	266 (56.5)	88 (51.8)	.43	.09	.19
Secondary transport from external hospital, *n* (%)	2,153 (44.5)	1,864 (44.5)	218 (46.3)	71 (41.8)	.45	.49	.57
Last-seen-well or symptom onset to hospital arrival (min), median (IQR)	173 (75–345)	172 (75–341)	187 (74–364)	177 (86–389)	.36	.37	.46
Last-seen-well or symptom onset to hospital arrival > 6h, *n* (%)	1,055 (24.0)	903 (23.8)	112 (25.5)	40 (26.3)	.44	.47	.59
**Baseline imaging**							
CT, *n* (%)	4,513 (94.5)	3,914 (94.5)	436 (93.2)	163 (97.6)	.25	.08	.10
MRI, *n* (%)	265 (5.5)	229 (5.5)	32 (6.8)	4 (2.4)			
Perfusion-based imaging, *n* (%)	2,595 (53.7)	2,298 (54.8)	217 (46.1)	80 (47.1)	**<.001**	**.047**	**<.001**
ASPECTS, median (IQR)	9 (7–10)	9 (7–10)	8 (7–10)	8 (7–10)	**<.001**	**<.01**	**<.001**
**Occlusion site**							
ICA-non-T, *n* (%)	548 (11.3)	475 (11.3)	50 (10.6)	23 (13.5)	**<.01**	.14	**<.01**
ICA-T, *n* (%)	645 (13.3)	550 (13.1)	67 (14.2)	28 (16.5)			
MCA M1 proximal, *n* (%)	1,316 (27.2)	1,122 (26.8)	145 (30.8)	49 (28.8)			
MCA M1 distal, *n* (%)	856 (17.7)	739 (17.6)	87 (18.5)	30 (17.6)			
MCA M2, *n* (%)	882 (18.2)	758 (18.1)	94 (20.0)	30 (17.6)			
ACA only, *n* (%)	43 (0.9)	40 (1.0)	2 (0.4)	1 (0.6)			
VA only, *n* (%)	37 (0.8)	34 (0.8)	3 (0.6)	0			
BA (±VA/PCA), *n* (%)	428 (8.9)	405 (9.7)	18 (3.8)	5 (2.9)			
PCA (±VA), *n* (%)	79 (1.6)	70 (1.7)	5 (1.1)	4 (2.4)			
**Treatment details**							
Thrombolysis, *n* (%)	2,408 (49.8)	2,066 (49.3)	258 (54.8)	84 (49.4)	**.02**	.97	.08
No of passes, median (IQR)	2 (1–3)	1 (1–3)	2 (1–3)	2 (1–4)	**<.001**	**<.001**	**<.001**
Vasospams, *n* (%)	151 (3.1)	115 (2.7)	25 (5.3)	11 (6.5)	**<.01**	**<.01**	**<.001**
Dissection/Perforation, *n* (%)	116 (2.4)	70 (1.7)	27 (5.7)	19 (11.2)	**<.001**	**<.001**	**<.001**
Clot migration/embolisation, *n* (%)	193 (4.0)	148 (3.5)	32 (6.8)	13 (7.6)	**<.001**	**<.01**	**<.001**
First pass success, *n* (%)	2,000 (43.9)	1,827 (46.2)	129 (29.0)	44 (26.8)	**<.001**	**<.001**	**<.001**
Successful recanalisation (mTICI 2b/3), *n* (%)	4,120 (86.6)	3,575 (86.7)	406 (87.3)	139 (82.7)	.73	.14	.30
Complete recanalisation (mTICI 3), *n* (%)	2,328 (49.0)	2,102 (51.0)	169 (36.3)	57 (33.9)	**<.001**	**<.001**	**<.001**
ICA stenosis > 70%, *n* (%)	600 (12.4)	499 (11.9)	76 (16.1)	25 (14.7)	**<.01**	.27	**.02**
Stenting, *n* (%)	439 (9.1)	364 (8.7)	53 (11.3)	22 (12.9)	.06	.06	**.04**

Abbreviations: ACA = anterior cerebral artery; aICH = asymptomatic ICH; ASPECTS = Alberta Stroke Program Early CT Score; BA = basilar artery; ICA = internal carotid artery; ICA-non-T = non-terminal ICA; ICA-T = terminal ICA; MCA: middle cerebral artery; mTICI = modified thrombolysis in cerebral infarction; PCA = posterior cerebral artery; VA = vertebral artery. *p* values <0.05 marked bold.

### Treatment details

Rates of thrombolysis were higher in patients with aICH than in patients with no ICH (54.8% vs 49.3%, *P* = .02). The number of passes was higher in patients with aICH (2 [1–3] vs 1 [1–3]), as was vasospasms (5.3% vs 2.7%), clot migration/embolisation (6.8% vs 3.5%) and dissection/perforation (5.7% vs 1.7%; *P* < .001 for all comparisons). While rates of successful recanalisation did not differ between patients with aICH (87.3%) and no ICH (86.7%), we found significantly lower rates of complete recanalisation in patients with aICH (36.3% vs 51.0%, *P* < .001). Further treatment details as well as data on patients with sICH can be found in Table 1.

### Clinical outcomes

When compared to patients with no ICH, patients with aICH had worse clinical outcome.

**Figure 1:**
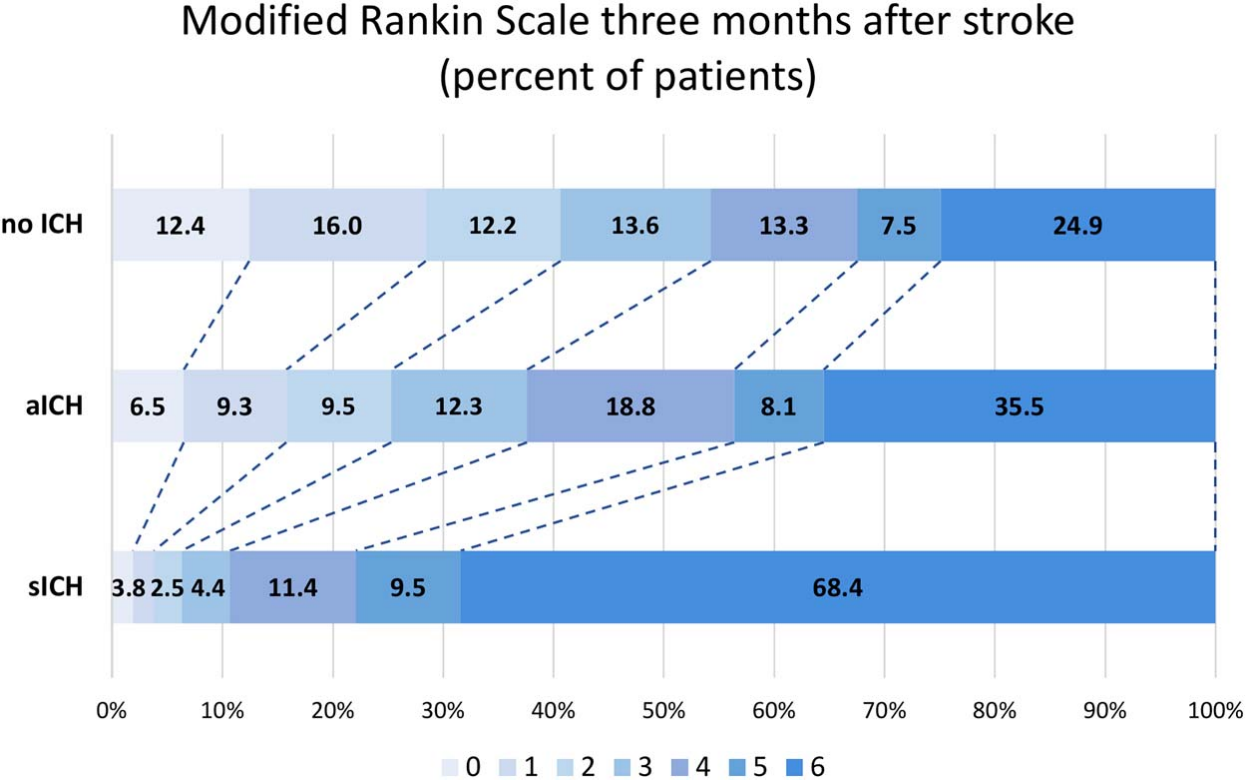
mRS at 3 months by ICH status.

Functional independence at 3 months (mRS ≤ 2) was reached by 25.4% of patients with aICH and 40.8% of patients with no ICH; adjusted odds ratio [aOR] 0.43 (95% CI, 0.32–0.58). Similarly, rates of fair outcome at 3 months (mRS ≤ 3) were lower in the presence of aICH (37.8% vs 54.4% [no ICH]; aOR 0.44 [95% CI, 0.33–0.58]). Overall disability (mRS shift) was less likely to be reduced by EVT in patients with aICH (adjusted common OR for reduced disability 0.51 [95% CI, 0.41–0.63]), and the odds for mortality at day 90 was nearly twice as high (aOR 1.90 [95% CI, 1.44–2.51]). Short-term outcomes such as ENI (30.1% vs 45.0%, aOR 0.47 [95% CI, 0.37–0.60]) and in-hospital mortality (21.6% vs 12.6%, aOR 2.11 [95% CI, 1.55–2.86]) were also worse in patients with aICH (when compared to no ICH). For details regarding mRS distribution and estimates for sICH, see Table 3 and Figure 1. In sensitivity analyses adjusting for number of passes and rates of complete recanalisation, aICH remained independently associated with lower odds of functional independence (mRS ≤ 2; aOR 0.53 [95% CI, 0.39–0.71]). Detailed effect estimates of all covariates included in the multivariable model are reported in Table S2 in the Supplementary material.

**Table 3:** Clinical outcomes.

**Variable**	**No ICH** ***n* = 4,139**	**aICH** ***n* = 471**	**sICH** ***n* = 170**	**aOR (95% CI)** **aICH vs no ICH**	***P***	**aOR (95% CI)** **sICH vs no ICH**	***P***
mRS ≤ 2 at d90, *n* **(%)**	1509 **(40.8)**	109 **(25.4)**	10 **(6.5)**	0.43 (0.32–0.58)	<.001	0.06 (0.03–0.14)	<.001
mRS at d90, median (IQR)	3 (1–5)	4 (2–6)	6 (5–6)	Adj. common OR for reduced disability 0.51 (0.41–0.63)		Adj. common OR for reduced disability 0.08 (0.05–0.12)	<.001
0, *n* (%)	461 (12.4)	28 (6.5)	3 (1.9)				
1, *n* (%)	595 (16.0)	40 (9.3)	3 (1.9)				
2, *n* (%)	453 (12.2)	41 (9.5)	4 (2.5)				
3, *n* (%)	505 (13.6)	53 (12.3)	7 (4.4)				
4, *n* (%)	494 (13.3)	81 (18.8)	18 (11.4)				
5, *n* (%)	279 (7.5)	35 (8.1)	15 (9.5)				
6, *n* (%)	926 (24.9)	153 (35.5)	108 (68.4)				
mRS ≤ 3 at d90, *n* **(%)**	215 **(54.4)**	162 **(37.8)**	17 **(11.0)**	0.44 (0.33–0.58)	<.001	0.06 (0.03–0.11)	<.001
Mortality at d90, *n* **(%)**	926 **(24.9)**	153 **(35.5)**	108 **(68.4)**	1.90 (1.44–2.51)	<.001	11.95 (7.52–19.00)	<.001
Early neurological improvement, *n* **(%)**	1,885 **(45.0)**	142 **(30.1)**	–	0.47 (0.37–0.60)	<.001	–	–
In-hospital mortality, *n* **(%)**	522 **(12.6)**	101 **(21.6)**	85 **(50.0)**	2.11 (1.55–2.86)	<.001	10.66 (7.05–16.12)	<.001

Abbreviations: aICH = asymptomatic ICH; END = early neurological deterioration.

Adjusted for age, sex, NIHSS at admission, pre-stroke dependency, i.v. thrombolysis, last-seen-well or symptom onset to hospital arrival, diabetes mellitus, arterial hypertension, atrial fibrillation, hyperlipidemia, active smoking, antiplatelets, oral anticocagulation, ASPECTS, successful recanalisation (mTICI 2b/3 yes vs no).

sICH as defined as in ECASS-III: (ICH together with END of ≥ 4 points on the NIHSS in the absence of any other event more likely to cause END).

Early neurological improvement: decrease of NIHSS of at least 4 points between admission and 24-hour follow-up or reaching an NIHSS of 0 at 24 hours.

## Discussion

To our knowledge, this study represents the largest and most detailed original research on asymptomatic ICH (aICH) following EVT to date. By leveraging data from a large national prospective stroke registry, we provide a comprehensive analysis of aICH subtypes, procedural factors and the impact of aICH on functional outcomes.^32^ We report 3 major findings: First, bleeding patterns differ between aICH and sICH, with patients with aICH having more haemorrhagic transformation and monocompartmental bleedings, while sICH are more often PH combined with bleeding outside the infarcted tissue. Second, rates of complete reperfusion (mTICI3) were lower, while procedural complications such as vasospasms, clot migration/embolisation and dissection/perforation were higher in patients with aICH when compared to no ICH. Third, aICH was associated with worse clinical outcomes, both regarding short-term (ENI) and long-term follow-up (mRS at 3 months). Our findings reinforce the notion that aICH is not a benign phenomenon but rather a distinct entity with potential clinical consequences.

### Procedural factors

While analysing adverse events during thrombectomy, we identified a complex interplay between procedural factors, reperfusion quality and vascular integrity in the development of aICH. Proximal occlusions (ICA-T/M1) were more prevalent in aICH than in patients with no ICH, and these patients also required a higher number of retrieval passes (median 2 vs 1). A greater clot burden and prolonged or more aggressive endovascular manipulation may contribute to vessel trauma, endothelial disruption and subsequent blood extravasation into brain parenchyma.^15^^,^^33^ While successful recanalisation rates were similar between aICH and no ICH groups, the significantly lower rates of complete recanalisation in patients with aICH (36.3% vs 51.0%) indicate that suboptimal reperfusion may facilitate delayed haemorrhagic changes (due to residual thrombus burden or persistent microvascular dysfunction). This finding is consistent with previous studies, which also described an association of incomplete reperfusion with both sICH and aICH.^15^^,^^20^^,^^33–36^ Notably, vasospasm during EVT was more common in patients with aICH (5.3% vs 2.7% [no ICH]), which may further contribute to suboptimal reperfusion by reducing cerebral blood flow, delaying tissue recovery and mimicking residual thrombus or intracranial stenosis.^37^^,^^38^ Additionally, the increased rate of clot migration/embolisation (6.8% vs 3.5%) and vessel dissection/perforation (5.7% vs 1.7%) in patients with aICH raises important considerations about the mechanical aspects of thrombectomy. Collectively, these findings suggest that a more aggressive or prolonged endovascular intervention, alongside vasospasm-related perfusion deficits, may amplify vascular injury and inflammatory responses, predisposing to aICH.^39^

### Bleeding details

A key strength of our study is the detailed classification of haemorrhagic subtypes, using the Heidelberg Bleeding Classification, which provides a nuanced view on aICH and the different ICH patterns in comparison to sICH. While parenchymal haematoma alone occurred at similar rates in aICH and sICH combined PH with haemorrhage outside infarcted tissue or other haemorrhage types (subarachnoid haemorrhage [SAH], IVH, subdural haemorrhage) was significantly more frequent in patients with sICH, similar to the findings reported by van der Steen.^20^ The presence of any multifocal bleeding across Heidelberg classes was also more frequent in sICH (34.1%) than aICH (20.2%). Furthermore, bleeding with intraventricular involvement occurred more frequently in sICH than in aICH (19.7% vs 6.8%). Given that haemorrhage volume and IVH are well-established predictors of poor functional outcome, the combination of multiple haemorrhagic components may contribute to the symptomatic presentation of ICH.^39^^,^^40^ Subarachnoid haemorrhage, on the other hand, was present in approximately 30% of both aICH and sICH cases, suggesting that SAH in isolation may not be a primary determinant of neurological deterioration. Of note, we observed substantial heterogeneity in Heidelberg Bleeding Classes among patients with aICH. This supports the notion that outcome may not be determined solely by the bleeding morphology, but also by factors such as haemorrhage size and location.^41^ If cortical areas crucial for cognition are affected, even minor haemorrhagic transformation (class 1a/b) may tip the balance from mRS 2 to 3 in elderly patients. Subcortical parenchymal haematoma (class 1c) on the other hand, may not worsen acute symptoms, but might critically affect motor recovery, when descending pathways are disrupted. Subarachnoid haemorrhage (class 3c) may further affect recovery through mechanisms of delayed cerebral ischaemia.^42^^,^^43^ As the Heidelberg Classification does not consider lesion location or bleeding volume, further research towards a more differentiated assessment of haemorrhagic transformation is warranted.

### Clinical outcome

Our study provides novel insights into the impact of aICH on *early* functional recovery. Patients with aICH experienced significantly lower rates of ENI compared to those with no ICH (30.1% vs 45.0%; aOR 0.47; 95% CI, 0.37–0.60), a finding that has not been previously described. The fact that patients with aICH were only half as likely to reach ENI highlights a fundamental issue with the concept of aICH: Intracranial bleeding does not need to manifest with neurological deterioration to exert a harmful effect: Even a delay in early recovery is clinically significant. At 3 months, patients with aICH were less than half as likely to achieve either functional independence (mRS ≤ 2) or fair outcome (mRS ≤ 3), when compared to patients with no ICH. The mortality rate was also markedly increased in patients with aICH (35.5%), positioning aICH as an intermediate-risk category between no ICH (24.9%) and sICH (68.4%). This distinction raises important mechanistic questions about whether aICH represents a marker of vascular integrity and subclinical neuroinflammation, contributing to a slower trajectory of neurological repair rather than causing direct mass effect. The significantly lower probability of ENI may be driven by factors like lesion burden, microvascular injury and/or reperfusion quality, which warrants further pathophysiological investigation.^44^

Our findings are corroborated by previous studies, which further reinforce the clinical relevance of aICH. A prior meta-analysis by Harker et al. reported a mortality OR of 1.72 (95% CI, 1.17–2.53; *P* = .005), which is similar to our study’s estimate of 1.90 (95% CI, 1.44–2.51), providing independent confirmation of our results.^10^ While differences in study designs, inclusion criteria and haemorrhage classification methods may explain minor discrepancies in outcome measures, poor functional outcome in the meta-analysis was associated with an OR of 2.17 (95% CI, 1.81–2.60; *P* < .0001),^10^ aligning with our findings that patients with aICH are less than half as likely to achieve functional independence compared to those with no ICH. Guasch-Jiménez et al. similarly reported aICH to be associated with worse functional outcome and higher mortality, describing mRS ≤ 2 in 21.2% of patients with aICH, which corresponds to our findings of 25.4% functional independence at 3 months.^45^

Despite growing contradictory evidence,^10^ aICH after EVT is still often perceived as an inconsequential imaging phenomenon in clinical practice. Given the association with impaired early and long-term recovery as well as increased mortality we report, our results corroborate previous evidence and strongly support the notion that aICH should not be overlooked in thrombectomy trials. The inclusion of any ICH as a safety outcome in the large core trial Endovascular Therapy in Acute Anterior Circulation Large Vessel Occlusive Patients with a Large Infarct Core (ANGEL-ASPECTS), and in particular the fact that this outcome was included even in the study abstract, suggests that a paradigm shift may be underway. Given the high rates of aICH in ANGEL-ASPECTS (49%) and the growing number of patients with EVT and large infarct core—aICH may gain even more importance in the future.^2^ Procedural refinements aimed at minimising thrombectomy passes, mitigating vasospasm and optimising periprocedural blood pressure management may help reduce the incidence and potential impact of aICH. There is also an urgent need for research into neuroprotective strategies that could counteract delayed neurotoxic effects associated with aICH-related vascular injury.^46^

Our study has several limitations: First, we present retrospective, observational data, which are prone to bias by indication and can only be hypothesis-generating. Second, the study population predominantly consisted of patients treated at university hospitals, which limits the generalisability of our findings. Third, ECASS-III sICH classification was not performed by a central adjudication committee, possibly leading to centre-specific bias. Fourth, there was no central imaging reading, thus, independent or blinded imaging assessment was not possible, which may have led to an underreporting of aICH. Fifth, we did not assess infarct or bleeding volume and were not able to adjust for this factor.

## Conclusion

ICH after EVT is associated with poorer functional recovery and increased mortality, even in the absence of early neurological deterioration. Given these findings, the term “asymptomatic ICH” warrants reconsideration. Any ICH, regardless of END, might be considered a relevant safety outcome, both in future thrombectomy trials and in clinical practice.

## Supplementary Material

aakaf009_aICH_are_associated_with_worse_clinical_outcome_Supplement_clean_290925

aakaf009_aICH_are_associated_with_worse_clinical_outcome_Supplement_clean_TableS2

## Data Availability

Anonymized data not published within this article will be made available by reasonable request from a qualified investigator.

## References

[1] Johnson CO, Nguyen M, Roth GA, et al. Global, regional, and national burden of stroke, 1990–2016: a systematic analysis for the Global Burden of Disease Study 2016. Lancet Neurol. 2019;18:439-458. 10.1016/S1474-4422(19)30034-1 30871944 PMC6494974

[2] Huo X, Ma G, Tong X, et al. Trial of endovascular therapy for acute ischemic stroke with large infarct. N Engl J Med. 2023;388:1272-1283. 10.1056/NEJMoa2213379 36762852

[3] Goyal M, Menon BK, van Zwam WH, et al. Endovascular thrombectomy after large-vessel ischaemic stroke: a meta-analysis of individual patient data from five randomised trials. Lancet. 2016;387:1723-1731. 10.1016/S0140-6736(16)00163-X 26898852

[4] Jovin TG, Nogueira RG, Lansberg MG, et al. Thrombectomy for anterior circulation stroke beyond 6 h from time last known well (AURORA): a systematic review and individual patient data meta-analysis. Lancet. 2022;399:249-258. 10.1016/S0140-6736(21)01341-6 34774198

[5] Albers GW, Marks MP, Kemp S, et al. Thrombectomy for stroke at 6 to 16 hours with selection by perfusion imaging. N Engl J Med. 2018;378:708-718. 10.1056/NEJMoa1713973 29364767 PMC6590673

[6] Nogueira RG, Jadhav AP, Haussen DC, et al. Thrombectomy 6 to 24 hours after stroke with a mismatch between deficit and infarct. N Engl J Med. 2018;378:11-21. 10.1056/NEJMoa1706442 29129157

[7] Yoshimura S, Sakai N, Yamagami H, et al. Endovascular therapy for acute stroke with a large ischemic region. N Engl J Med. 2022;386:1303-1313. 10.1056/NEJMoa2118191 35138767

[8] Sarraj A, Hassan AE, Abraham MG, et al. Trial of endovascular thrombectomy for large ischemic strokes. N Engl J Med. 2023;388:1259-1271. 10.1056/NEJMoa2214403 36762865

[9] Huo X, Ma G, Tong X, et al. Trial of endovascular therapy for acute ischemic stroke with large infarct. N Engl J Med. 2023;388:1272-1283. 10.1056/NEJMoa2213379 36762852

[10] Harker P, Aziz YN, Vranic J, et al. Asymptomatic intracerebral hemorrhage following endovascular stroke therapy is not benign: a systematic review and meta-analysis. J Am Heart Assoc. 2024;13:31749. 10.1161/JAHA.123.031749 PMC11010099 38348800

[11] Kent DM, Hinchey J, Price LL, Levine SR, Selker HP. In acute ischemic stroke, are asymptomatic intracranial hemorrhages clinically innocuous? Stroke. 2004;35:1141-1146. 10.1161/01.STR.0000125712.02090.6e 15087567

[12] Yoon W, Jung MY, Jung SH, Park MS, Kim JT, Kang HK. Subarachnoid hemorrhage in a multimodal approach heavily weighted toward mechanical thrombectomy with solitaire stent in acute stroke. Stroke. 2013;44:414-419. 10.1161/STROKEAHA.112.675546 23287788

[13] Yilmaz U, Walter S, Körner H, et al. Peri-interventional subarachnoid hemorrhage during mechanical thrombectomy with stent retrievers in acute stroke: a retrospective case-control study. Clin Neuroradiol. 2015;25:173-176. 10.1007/s00062-014-0294-6 24526101

[14] Luff MK, Khezri N, Miralbes S, et al. Hemorrhagic transformation in acute ischemic stroke: hemorrhagic subtypes and symptomatic intracranial hemorrhage. J Neurointerv Surg. 2025;0:1-10. 10.1136/jnis-2024-021725 PMC12322388 38969497

[15] Nawabi J, Kniep H, Broocks G, et al. Clinical relevance of asymptomatic intracerebral hemorrhage post thrombectomy depends on angiographic collateral score. J Cereb Blood Flow Metab. 2020;40:1599-1607. 10.1177/0271678X19871253 31433715 PMC7370359

[16] Hao Y, Liu W, Wang H, et al. Prognosis of asymptomatic intracranial hemorrhage after endovascular treatment. J Neurointerv Surg. 2019;11:123-126. 10.1136/neurintsurg-2018-013848 29970621

[17] Constant Dit Beaufils P, Preterre C, De Gaalon S, et al. Prognosis and risk factors associated with asymptomatic intracranial hemorrhage after endovascular treatment of large vessel occlusion stroke: a prospective multicenter cohort study. Eur J Neurol. 2021;28:229-237. 10.1111/ene.14539 32935401

[18] Jiang F, Zhao W, Wu C, et al. Asymptomatic intracerebral hemorrhage may worsen clinical outcomes in acute ischemic stroke patients undergoing thrombectomy. J Stroke Cerebrovasc Dis. 2019;28:1752-1758. 10.1016/j.jstrokecerebrovasdis.2019.02.006 30926220

[19] Neuberger U, Kickingereder P, Schönenberger S, et al. Risk factors of intracranial hemorrhage after mechanical thrombectomy of anterior circulation ischemic stroke. Neuroradiology. 2019;61:461-469. 10.1007/s00234-019-02180-6 30778621

[20] Van Der Steen W, Van Der Ende NAM, Luijten SPR, et al. Type of intracranial hemorrhage after endovascular stroke treatment: association with functional outcome. J Neurointerv Surg. 2023;15:971-976. 10.1136/jnis-2022-019474 36261280 PMC10511981

[21] Ohashi SN, Delong JH, Kozberg MG, et al. Role of inflammatory processes in hemorrhagic stroke. Stroke. 2023;54:605-619. 10.1161/STROKEAHA.122.037155 36601948

[22] Alegiani AC, Dorn F, Herzberg M, et al. Systematic evaluation of stroke thrombectomy in clinical practice: the German Stroke Registry Endovascular Treatment. Int J Stroke. 2019;14:372-380. https://pubmed.ncbi.nlm.nih.gov/30346260/, 10.1177/1747493018806199 30346260

[23] Riegler C, Rücker V, von Rennenberg R, et al. Time trends in mechanical thrombectomy (2017–2021): do real-world data reflect advances in evidence? Front Neurol. 2025;15:1517276. 10.3389/fneur.2024.1517276 40008260 PMC11850263

[24] von Kummer R, Broderick JP, Campbell BCV, et al. The Heidelberg Bleeding Classification. Stroke. 2015;46:2981-2986. 10.1161/STROKEAHA.115.010049 26330447

[25] Hacke W, Kaste M, Bluhmki E, et al. Thrombolysis with alteplase 3 to 4.5 hours after acute ischemic stroke. N Engl J Med. 2008;359:1317-1329. 10.1056/NEJMoa0804656 18815396

[26] Rangaraju S, Haussen D, Nogueira RG, Nahab F, Frankel M. Comparison of 3-month stroke disability and quality of life across modified Rankin scale categories. Interv Neurol. 2017;6:36-41. 10.1159/000452634 28611832 PMC5465722

[27] Nagaraja N, Warach S, Hsia AW, et al. Association between neurologic improvement with decline in blood pressure and recanalization in stroke. JAMA Neurol. 2014;71:1555. 10.1001/jamaneurol.2014.2036 25330362 PMC5257035

[28] Dargazanli C, Consoli A, Barral M, et al. Impact of modified TICI 3 versus modified TICI 2b reperfusion score to predict good outcome following endovascular therapy. Am J Neuroradiol. 2017;38:90-96. 10.3174/ajnr.A4968 27811134 PMC7963649

[29] IBM . How to cite IBM SPSS Statistics or earlier versions of SPSS [Internet] [cited 26.02.2025]. https://www.ibm.com/support/pages/how-cite-ibm-spss-statistics-or-earlier-versions-spss

[30] Alegiani AC, Dorn F, Herzberg M, et al. Systematic evaluation of stroke thrombectomy in clinical practice: the German Stroke Registry Endovascular Treatment. Int J Stroke. 2019;372-380. 10.1177/1747493018806199 30346260

[31] Wollenweber FA, Tiedt S, Alegiani A, et al. Functional outcome following stroke thrombectomy in clinical practice. Stroke. 2019;50:2500-2506. 10.1161/STROKEAHA.119.026005 31337298

[32] German Stroke Registry [Internet] [cited 26.02.2025]. https://www.german-stroke-registry.de/german-stroke-registry

[33] Yogendrakumar V, Al-Ajlan F, Najm M, et al. Clot burden score and early ischemia predict intracranial hemorrhage following endovascular therapy. AJNR Am J Neuroradiol. 2019;40:655-660. 10.3174/ajnr.A6009 30872416 PMC7048514

[34] Kang Z, Liu G, Fan R, et al. Prognosis and prediction of asymptomatic intracranial hemorrhage after endovascular thrombectomy: a multi-center study. J Endovasc Ther. 2023;32:1724-1735. 10.1177/15266028231219990 38149437

[35] van Landeghem N, Ziegenfuß C, Demircioglu A, et al. Impact of post-thrombectomy isolated subarachnoid hemorrhage on neurological outcomes in patients with anterior ischemic stroke—a retrospective single-center observational study. Neuroradiology. 2024;66:1737-1745. 10.1007/s00234-024-03424-w 38980345 PMC11424715

[36] Winkelmeier L, Faizy TD, Brekenfeld C, et al. Comparison of thrombolysis in cerebral infarction (TICI) 2b and TICI 3 reperfusion in endovascular therapy for large ischemic anterior circulation strokes. J Neurointerv Surg. 2024;16:1076-1082. 10.1136/jnis-2023-020724 37777256 PMC11503081

[37] Mosimann PJ, Kaesmacher J, Gautschi D, et al. Predictors of unexpected early reocclusion after successful mechanical thrombectomy in acute ischemic stroke patients. Stroke. 2018;49:2643-2651. 10.1161/STROKEAHA.118.021685 30355192

[38] Pilgram-Pastor SM, Piechowiak EI, Dobrocky T, et al. Stroke thrombectomy complication management. J Neurointerv Surg. 2021;13:912. 10.1136/neurintsurg-2021-017349 34158401 PMC8458081

[39] Steiner T, Diringer MN, Schneider D, et al. Dynamics of intraventricular hemorrhage in patients with spontaneous intracerebral hemorrhage: risk factors, clinical impact, and effect of hemostatic therapy with recombinant activated factor VII. Neurosurgery. 2006;59:767-773. 10.1227/01.NEU.0000232837.34992.32 17038942

[40] Yogendrakumar V, Ramsay T, Fergusson D, et al. New and expanding ventricular hemorrhage predicts poor outcome in acute intracerebral hemorrhage. Neurology. 2019;93:e879. 10.1212/WNL.0000000000008007 31371565 PMC6745728

[41] Teo KC, Fong SM, Leung WCY, et al. Location-specific hematoma volume cutoff and clinical outcomes in intracerebral hemorrhage. Stroke. 2023;54:1548-1557. 10.1161/STROKEAHA.122.041246 37216445 PMC10266339

[42] Dodd WS, Laurent D, Dumont AS, et al. Pathophysiology of delayed cerebral ischemia after subarachnoid hemorrhage: a review. J Am Heart Assoc. 2021;10:21845. 10.1161/JAHA.121.021845 PMC8475656 34325514

[43] Tschoe C, Bushnell CD, Duncan PW, Alexander-Miller MA, Wolfe SQ. Neuroinflammation after intracerebral hemorrhage and potential therapeutic targets. J Stroke. 2020;22:29. 10.5853/jos.2019.02236 32027790 PMC7005353

[44] Guasch-Jiménez M, Díaz GE, Lambea-Gil Á, et al. Influence of asymptomatic hemorrhagic transformation after endovascular treatment on stroke outcome a population-based study. Neurology. 2025;104:e213509. 10.1212/WNL.0000000000213509 40228188

[45] Hukamdad M, Biller J, Testai FD, Trifan G. Endovascular thrombectomy for large core volume acute ischemic stroke. Updated systematic review and meta-analysis: thrombectomy for large core acute ischemic strokes. J Stroke Cerebrovasc Dis. 2025;34:108135. 10.1016/j.jstrokecerebrovasdis.2024.108135 39537044

[46] Haupt M, Gerner ST, Bähr M, Doeppner TR. Neuroprotective strategies for ischemic stroke—future perspectives. Int J Mol Sci. 2023;24:4334. 10.3390/ijms24054334 36901765 PMC10002358

