# Supplementary material for: Intracranial haemorrhage without early clinical deterioration after mechanical thrombectomy: rethinking the “asymptomatic” label
Source: Eur Stroke J. 2026 Jan 1;11(1):aakaf009. doi: 10.1093/esj/aakaf009 (PMC12866645; doi:10.1093/esj/aakaf009)
Supplement: aakaf009_aICH_are_associated_with_worse_clinical_outcome_Supplement_clean_290925 [file aakaf009_aich_are_associated_with_worse_clinical_outcome_supplement_clean_290925.docx]

| **Table S1: Detailed Heidelberg Bleeding Classification** | | | | |
| --- | --- | --- | --- | --- |
|  | All  N=641 | aICH  n=471 | sICH  n=170 |  |
| **Follow-Up Imaging** | | | | |
| CT – n (%) | 554 **(86.4)** | 392 **(83.2)** | 162 **(95.3)** | **<0.001** |
| MRI – n (%) | 87 **(13.6)** | 79 **(16.8)** | 8 **(4.7)** |  |
| **Heidelberg Bleeding Classification** | | | | |
| Hemorrhagic transformation  (HT) | 200 **(31.2)** | 163 **(34.6)** | 37 **(21.8)** | **<0.01** |
| Class 1a | 100 **(15.6)** | 83 **(17.6)** | 17 **(10.0)** | **0.02** |
| Class 1b | 100 **(15.6**) | 80 **(17.0)** | 20 **(11.8)** | 0.11 |
| Parenchymal hematoma  (PH; any) | 245 **(38.2)** | 163 **(34.6)** | 82 **(48.2)** | **<0.01** |
| Parenchymal hematoma (only) | 155 **(24.3)** | 112 **(23.9)** | 43 **(25.3)** | 0.71 |
| Class 1c | 76 **(11.9)** | 62 **(13.2)** | 14 **(8.2)** | 0.09 |
| Class 2 | 67 **(10.5)** | 42 **(8.9)** | 25 **(14.7)** | **0.03** |
| Class 3a | 12 **(1.9)** | 8 **(1.7)** | 4 **(2.4)** | 0.59 |
| Parenchymal hematoma plus (any 3a/b/c/d) | 90 **(14.0)** | 51 **(10.8)** | 39 **(22.9)** | **<0.001** |
| Class 1c plus | 26 **(4.1)** | 19 **(4.0)** | 7 **(4.1)** | 0.96 |
| Class 1c + 3a +3c | 1 **(0.1)** | 1 **(0.2)** | 0 | 0.55 |
| Class 1c + 3b | 4 **(0.6)** | 3 **(0.6)** | 1 **(0.6)** | 0.95 |
| Class 1c +3b + 3c | 3 **(0.5)** | 3 **(0.6)** | 0 | 0.30 |
| Class 1c + 3c | 18 **(2.8)** | 12 **(2.5)** | 6 **(3.5)** | 0.51 |
| Class 2 plus  (any 3a/b/c/d) | 57 **(8.9)** | 28 **(5.9)** | 29 **(17.1)** | **<0.001** |
| Class 2 + 3a/3b/3c | 2 **(0.3)** | 1 **(0.2)** | 1 **(0.6)** | 0.45 |
| Class 2 + 3a/3b/3d | 1 **(0.2)** | 0 | 1 **(0.56)** | 0.10 |
| Class 2 + 3b | 11 **(1.7)** | 6 **(1.3)** | 5 **(2.9)** | 0.15 |
| Class 2 + 3b/3c | 21 **(3.3)** | 8 **(1.7)** | 13 **(7.6)** | **<0.001** |
| Class 2 + 3b/3c/3d | 1 **(0.2)** | 1 **(0.2)** | 0 | 0.55 |
| Class 2 +3c | 19 **(3.0)** | 12 **(2.5)** | 7 **(4.1)** | 0.30 |
| Class 2 + 3c/3d | 2 **(0.3)** | 0 | 2 **(1.3)** | **0.02** |
| Class 3a plus | 7 **(1.1)** | 4 **(0.8)** | 3 **(1.8)** | 0.33 |
| Class 3a + 1b | 1 **(0.2)** | 0 | 1 **(0.6)** | 0.10 |
| Class 3a + 1b + 3b/c | 1 **(0.2)** | 1 **(0.2)** | 0 | 0.55 |
| Class 3a + 3c | 5 **(0.8)** | 3 **(0.6)** | 2 **(1.2)** | 0.49 |
| Intraventricular hemorrhage (IVH; 3b) | 5 **(0.8)** | 3 **(0.6)** | 2 **(1.2)** | 0.49 |
| 3b only | 1 **(0.1)** | 1 **(0.2)** | 0 | 0.55 |
| 3b + 1a | 1 **(0.2)** | 0 | 1 **(0.6)** | 0.10 |
| 3b + 1b | 3 **(0.5)** | 2 **(0.4)** | 1 **(0.6)** | 0.79 |
| Subarachnoid hemorrhage (SAH; 3c; any) | 189 **(29.5)** | 140 **(29.7)** | 49 **(28.8)** | 0.83 |
| 3c (only) | 129 **(20.1)** | 97 **(20.6)** | 32 **(18.8)** | 0.62 |
| 3c plus | 60 **(9.4)** | 43 **(9.1)** | 17 **(10.0)** | 0.74 |
| 3c + 1a | 14 **(2.2)** | 11 **(2.3)** | 3 **(1.8)** | 0.66 |
| 3c + 1b | 26 **(4.1)** | 21 **(4.5)** | 5 **(2.9)** | 0.39 |
| 3c + 3b | 13 **(2.0)** | 6 **(1.3)** | 7 **(4.1)** | **0.02** |
| 3c +3b + 1a | 3 **(0.5)** | 3 **(0.6)** | 0 | 0.26 |
| 3c + 3b +1b | 3 **(0.5)** | 1 **(0.2)** | 2 **(1.2)** | 0.11 |
| 3c + 3d | 1 **(0.2)** | 1 **(0.2)** | 0 | 0.55 |
| Subdural hemorrhage  (SDH; 3d) | 2 **(0.3)** | 2 **(0.4)** | 0 | 0.40 |
| *More than one class – n (%)* | 153 **(23.9)** | 95 **(20.2)** | 58 **(34.1)** | **<0.001** |
| *Any IVH*  *(+/- HI, PH or SAH)* | 68 **(10.6)** | 36 **(7.6)** | 32 **(18.8)** | **<0.001** |

| **Table S2: Sensitivity analysis: Factors associated with functional independence**  **(mRS ≤ 2) at three months** | | |
| --- | --- | --- |
| Variable | aOR (95%-CI) | p |
| aICH (vs. no ICH) | 0.53 (0.39 – 0.71) | <0.001 |
| Complete Recanalization (mTICI 3) | 1.79 (1.48 – 2.17) | <0.001 |
| No of passes (per +1 pass) | 0.80 (0.75 – 0.85) | <0.001 |
| Age (per +1 year) | 0.94 (0.93 – 0.95) | <0.001 |
| Male (vs. female) | 1.37 (1.13 – 1.65) | <0.01 |
| NIHSS at admission (per +1 point) | 0.89 (0.88 – 0.91) | <0.001 |
| Pre-Stroke mRS ≤ 2 | 10.93 (6.66 – 17.95) | <0.001 |
| Arterial Hypertension | 0.69 (0.55 – 0.86) | <0.01 |
| Diabetes mellitus | 0.56 (0.45 – 0.71) | <0.001 |
| Hyperlipidemia | 1.11 (0.92 – 1.35) | 0.28 |
| Active Smoking | 0.76 (0.58 – 1.00) | 0.05 |
| Atrial Fibrillation | 1.04 (0.84 – 1.30) | 0.71 |
| Antiplatelets at basline | 0.76 (0.61 – 0.96) | 0.02 |
| Oral Anticoagulation at baseline | 0.74 (0.56 – 0.98) | 0.04 |
| Time from Last-Seen-Well (or symptom onset) to hospital admission (per +30 minutes) | 0.98 (0.98 – 0.99) | <0.01 |
| ASPECTS (per +1 point) | 1.17 (1.11 – 1.24) | <0.001 |
| Intravenous Thrombolysis | 1.36 (1.09 – 1.69) | <0.01 |
