# Supplementary material for: Intracranial haemorrhage without early clinical deterioration after mechanical thrombectomy: rethinking the “asymptomatic” label
Source: Eur Stroke J. 2026 Jan 1;11(1):aakaf009. doi: 10.1093/esj/aakaf009 (PMC12866645; doi:10.1093/esj/aakaf009)
Supplement: aakaf009_aICH_are_associated_with_worse_clinical_outcome_Supplement_clean_TableS2 [file aakaf009_aich_are_associated_with_worse_clinical_outcome_supplement_clean_tables2.docx]

| **Table S2: Sensitivity analysis: Factors associated with functional independence**  **(mRS ≤ 2) at three months** | | |
| --- | --- | --- |
| Variable | aOR (95%-CI) | p |
| aICH (vs. no ICH) | 0.53 (0.39 – 0.71) | <0.001 |
| Complete Recanalization (mTICI 3) | 1.79 (1.48 – 2.17) | <0.001 |
| No of passes (per +1 pass) | 0.80 (0.75 – 0.85) | <0.001 |
| Age (per +1 year) | 0.94 (0.93 – 0.95) | <0.001 |
| Male (vs. female) | 1.37 (1.13 – 1.65) | <0.01 |
| NIHSS at admission (per +1 point) | 0.89 (0.88 – 0.91) | <0.001 |
| Pre-Stroke mRS ≤ 2 | 10.93 (6.66 – 17.95) | <0.001 |
| Arterial Hypertension | 0.69 (0.55 – 0.86) | <0.01 |
| Diabetes mellitus | 0.56 (0.45 – 0.71) | <0.001 |
| Hyperlipidemia | 1.11 (0.92 – 1.35) | 0.28 |
| Active Smoking | 0.76 (0.58 – 1.00) | 0.05 |
| Atrial Fibrillation | 1.04 (0.84 – 1.30) | 0.71 |
| Antiplatelets at baseline | 0.76 (0.61 – 0.96) | 0.02 |
| Oral Anticoagulation at baseline | 0.74 (0.56 – 0.98) | 0.04 |
| Time from Last-Seen-Well (or symptom onset) to hospital admission (per +30 minutes) | 0.98 (0.98 – 0.99) | <0.01 |
| ASPECTS (per +1 point) | 1.17 (1.11 – 1.24) | <0.001 |
| Intravenous Thrombolysis | 1.36 (1.09 – 1.69) | <0.01 |
